# An Atypical Case of Chilaiditi Syndrome

**DOI:** 10.7759/cureus.10815

**Published:** 2020-10-05

**Authors:** Gillean Cortes, Riya Kulkarni, Nida Hasan, Korinn Vandervall, Mark M Aloysius

**Affiliations:** 1 Medicine, A.T. Still University School of Osteopathic Medicine in Arizona, Mesa, USA; 2 Internal Medicine, The Wright Center for Graduate Medical Education, Scranton, USA

**Keywords:** chilaiditi sign, chilaiditi syndrome, pneumoperitoneum

## Abstract

Chilaiditi's sign is a rare radiological finding in which a portion of the colon or small intestine is interposed between the liver and right hemidiaphragm. We present a 28-year-old male who came to the emergency room with nausea and vomiting. A computed tomography scan without contrast of the abdomen and pelvis showed a new focus of air in the perihepatic region, suggesting a pneumoperitoneum or a loop of bowel. Exploratory laparotomy was pursued but deferred after a multi-disciplinary review of the imaging. A decision was made to pursue conservative management with a diagnosis of Chilaiditi syndrome. This case illustrates the importance of maintaining a broad differential when approaching a patient with abdominal distress and possible pneumoperitoneum, especially when the clinical picture does not align with radiological findings. Early consideration of Chilaiditi syndrome is important to minimize unnecessary surgical intervention such as laparotomy or further endoscopic intervention, which may lead to potential complications such as perforation, bowel wall ischemia, or respiratory failure.

## Introduction

Chilaiditi sign is a rare radiological finding in which a portion of the colon or small intestine is interposed between the liver and right hemidiaphragm. It rarely causes symptoms but when patients present with gastrointestinal symptoms such as abdominal pain, nausea or vomiting, and/or respiratory distress and chest pain, Chilaiditi syndrome is established.

## Case presentation

We present a 28-year-old male who came to the emergency room with nausea and vomiting associated with sudden onset, intermittent, sharp, midline abdominal pain with no aggravating or alleviating factors. He denied fever, cough, chest pain, shortness of breath, diarrhea, constipation, heartburn, dysuria, any trauma or prior surgeries.

Upon physical examination, he was afebrile, hemodynamically stable, and in mild distress due to pain. The abdomen was soft, non-distended with tenderness in the epigastric and umbilical regions. Bowel sounds were normal in all quadrants. There was no rebound tenderness, rigidity, or involuntary guarding. No palpable abdominal masses were appreciated.

**Figure 1:**
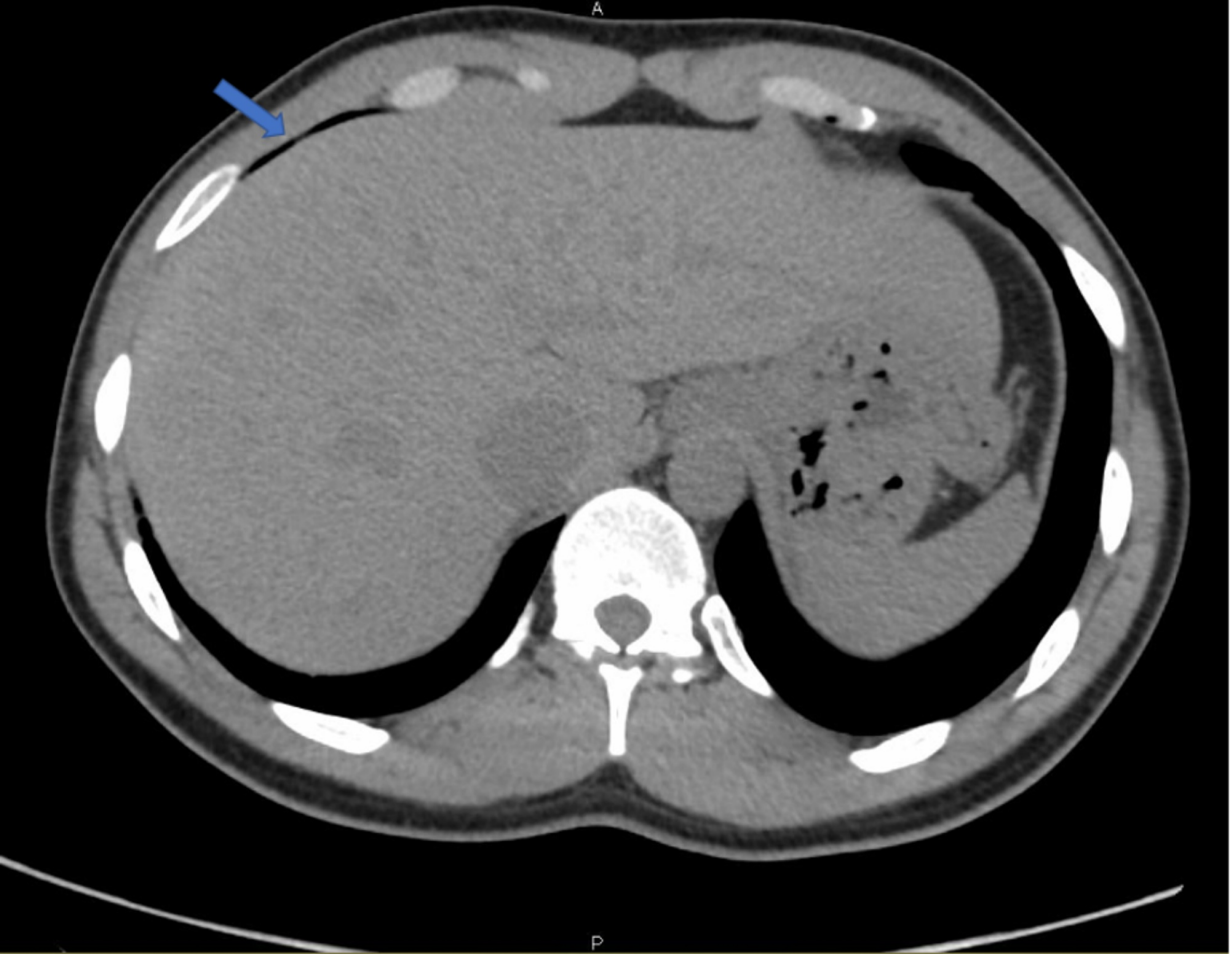
CT scan of the abdomen showing free air in the perihepatic region CT: computed tomography

Laboratory values were only significant for hypokalemia (3.3 mmol/L) and lactic acidosis (2.4 mmol/L) but otherwise unremarkable for an infectious presentation with normal white blood cell counts and liver function tests. Transabdominal ultrasound was unremarkable. A computed tomography (CT) scan without contrast of the abdomen and pelvis showed a new focus of air in the perihepatic region, suggesting a tiny pneumoperitoneum or loop of bowel (Figure 1).

The patient was stable and without any evidence of bowel obstruction, appendicitis, or inflammatory bowel disease. A CT scan of the chest also ruled out pneumothorax. Surgery was consulted and the chance of bowel perforation, cholelithiasis and cholecystitis were excluded. Exploratory laparotomy was pursued but deferred after a multi-disciplinary review of the imaging. A decision was made to pursue conservative management with a diagnosis of Chilaiditi syndrome. He was admitted two days later in which the patient’s vitals remained stable throughout the hospital course after being treated supportively with intravenous fluids and bowel rest. The patient reported significant improvement with no new complaints and regular diet resumed. He was discharged from the hospital the next day.

## Discussion

Demetrius Chilaiditi in 1910 first described a series of three cases with interposition of the colon or small intestine between the liver and hemidiaphragm, a finding now called Chilaiditi’s sign [1]. Risk factors include liver or colon abnormalities such as increased colonic mobility, chronic constipation, lax suspensory ligaments or complications of post-procedural interventions [2,3]. Chilaiditi syndrome is a manifestation of symptoms in addition to Chilaiditi’s sign. The very infrequent prevalence of Chilaiditi syndrome makes it a difficult diagnosis as it can be mistaken for pneumoperitoneum amongst other diagnoses. The incidence is reported to be 0.025% to 0.28% and is four times more common in males than females [4]. Commonly associated clinical symptoms of Chilaiditi syndrome are respiratory distress, angina like chest pain, anorexia, abdominal pain, vomiting, constipation, intestinal obstruction, and volvulus [5,6].

It is important to consider Chilaiditi syndrome as a differential in a patient with abdominal distress where the clinical picture does not align with radiological findings. Chilaiditi syndrome can present differently from one patient to another. Because Chilaiditi syndrome is rare, it is critical for clinicians to use radiological imaging modalities such as chest and abdominal radiographs, CT scan, and ultrasound to rule out other potential causes of pneumoperitoneum of which Chilaiditi syndrome can mimic [6,7]. Recognition of Chilaiditi syndrome can minimize unnecessary surgical intervention such as laparotomy as in this patient. This can lead to further preventable possible consequences such as hemorrhage, infection, and adhesions [8]. Endoscopic evaluation such as colonoscopies should be avoided in patients with Chilaiditi syndrome as the interposed bowel could be high risk for bowel perforation [9]. Although generally asymptomatic, late recognition of Chilaiditi syndrome can lead to detrimental consequences such as bowel obstruction, volvulus, bowel ischemia, and even perforation [5,10].

Treatment is usually conservative and includes bowel rest, intravenous fluids, nasogastric tube decompression, and laxatives. If conservative measures fail, there may be need for surgical intervention that could range from simple measures such as colonoscopic reduction to complex surgeries such as colonic resection [2]. Overall, considering Chilaiditi syndrome in patients with potential discordance in clinical symptomatology, radiological evidence, and rapid conservative treatment of this condition can lead to a significant change in hospital course. Diagnosing this condition early can lead to reduced hospital stay and potential complications such as hospital-acquired infections, financial burdens, and overall hospital-associated mortality.

## Conclusions

Chilaiditi syndrome is a rare manifestation that can present atypically. This case illustrates the importance of maintaining a broad differential when approaching a patient with possible pneumoperitoneum, especially when there is a significant clinical and diagnostic discrepancy. Early consideration of Chilaiditi syndrome is important to minimize unnecessary surgical intervention as it can be treated conservatively.
